# Editorial: Implications of immune landscape in tumor microenvironment

**DOI:** 10.3389/fimmu.2024.1491644

**Published:** 2024-09-20

**Authors:** Somchai Chutipongtanate, Selvarangan Ponnazhagan, Juana Serrano-López

**Affiliations:** ^1^ Milk, Microbiome, Immunity and Lactation Research for Child Health (MILCH) and Novel Therapeutics Lab, Division of Epidemiology, Department of Environmental and Public Health Sciences, University of Cincinnati College of Medicine, Cincinnati, OH, United States; ^2^ Department of Environmental and Public Health Sciences, University of Cincinnati College of Medicine, Cincinnati, OH, United States; ^3^ O’Neal Comprehensive Cancer Center, University of Alabama at Birmingham, Birmingham, AL, United States; ^4^ Department of Pathology, University of Alabama at Birmingham, Birmingham, AL, United States; ^5^ Experimental Hematology Lab, IIS-Fundación Jiménez Díaz, Universidad Autónoma de Madrid (UAM), Madrid, Spain

**Keywords:** tumor-immune stromal landscape, tumor microenvironment, next generation tools, immune checkpoint blockade, epigenetic mechanisms regulating immune suppression, ILINCS

The tumor microenvironment (TME) plays an essential role in cancer development, acting as a complex ecosystem where interactions between tumor cells and the neighboring stromal and immune components critically influence tumor progression, immune evasion, and therapeutic resistance. High-throughput technologies based on RNA sequencing are facilitating a picture of the tumor-derived oncotranscriptomes. That massive information is being used to generate new hypothesis for *in silico* screening of small molecules by for example, integrative LINCs (iLINCs) connectivity map or DREIMT platforms (1, 2). This Research Topic compiles a series of cutting-edge studies that provide detailed insights into the cellular and molecular mechanisms governing these interactions within the TME in several solid tumors. This Research Topic underscores the importance of targeting the TME in the development of next-generation cancer therapies. Firstly, Molina et al. underscores prognostic significance of tumor-infiltrating lymphocytes (TILs) in prostate cancer. By analyzing the density and phenotype of regulatory T cells (Tregs; CD3^+^Foxp3^+^) and memory T cells (Tmem; CD3^+^CD45RO^+^) within different tumor localizations, the study reveals that high Treg infiltration correlates with poor outcomes, while high Tmem infiltration is protective. Furthermore, they found that Foxp3 expression is highly associated with CTLA-4 and TIM-3 gene expression suggesting a potential immunosuppressive network at play. This finding highlighted the therapeutic promise of targeting Tregs in prostate cancer. Similarly, myeloid cells with immune suppressive functions, have also been involved in prostate cancer progression. Kobayashi et al. underscores the prognostic significance of myeloid-derived suppressor cell (MDSC) subtypes in prostate cancer. Elevated levels of PMN-MDSCs (CD33^+^HLA-DR^-^CD14-CD15^+^) subtype instead M-MDSC (CD33^+^HLA-DR^+^CD14^+^CD15^-^) correlate with poorer survival outcomes in the metastatic Castration-resistant prostate cancer (mCRPC) patients, making them potential biomarkers for prognosis and as therapeutic targets. This study emphasizes the need for further research into MDSCs and their role in cancer progression, particularly in identifying biomarkers for patient stratification and therapeutic interventions. Another myeloid cells affected by TME are Dendritic Cells (DCs), characterized by their crucial role in anti-tumor immunity. In blood cancers, the function of DCs is impaired due to the influence of the TME, leading them to remain in an immature state. However, isolating these cells and maturing them *in vitro* for use in vaccines may enhance treatment outcomes for cancer patients (3). In this topic Xiao et al. elucidate mechanisms by which the TME disrupts DC function and the potential therapeutic strategies to restore DC activity. Combining DC-based vaccines with immune checkpoint inhibitors and targeting the immunosuppressive TME could enhance the effectiveness of immunotherapies. Shifting focus to Pancreatic Ductal Adenocarcinoma (PDAC), one of the most lethal cancers due to its “immunologically cold” TME, the review by Joseph et al. provides a comprehensive analysis of the TME in PDAC. They emphasize the roles of Tumor Associated Macrophages (TAMs), MDSCs, and Tregs. Therapeutic strategies that target the stroma and modulate the immune response, such as combining immune checkpoint inhibitors with stroma-targeting agents, hold promise for improving outcomes in PDAC. Building on this, Freeman et al. have found that blocking Insulin-like Growth Factors (IGF) signaling increases production of CXCL9/10 by down-regulating AKT/phosphoSTAT3 in TAMs and Fibroblasts, thereby facilitating CD8^+^ Cytotoxic T Cell recruitment into Pancreatic Tumors. However, despite the increased infiltration, these CD8^+^ T cells remain functionally inactive, as shown by a GZMB assay. This study opens new avenues for combining IGF inhibitors with therapies that activate T cells, offering hope for overcoming the immunosuppressive TME in PDAC. The challenges of treating cancer are further complicated when it metastasizes to the bone, where a unique interplay between immune cells, bone cells, and tumor cells creates a complex therapeutic landscape. Chen et al. discusses how tumor-infiltrating cells, particularly from breast cancer, lung or prostate cancer, disarm cytotoxic T lymphocytes (CTL) and natural killer (NK) cells by upregulating programmed death-ligand 1 (PD-L1) expression or reprogramming bone cell toward osteoclastogenesis and consequently tumor progression. The study also highlights the importance of understanding tumor dormancy and the need for combination therapies to address the multifaceted nature of bone metastasis. In children, adolescents and young adults, osteosarcoma is the most common bone tumor. Orrapin et al. provides an in-depth review of the complex tumor-immune microenvironment (TIME) in osteosarcoma and the complex interactions within the TIME that drive tumor progression and resistance to therapy. Glioblastoma (GBM), a highly aggressive brain tumor with limited treatment options, presents another formidable challenge. Kushihara et al. investigates the immune microenvironment of GBM, revealing that tumors with high expression of O6-methylguanine-DNA (MGMT-H) exhibit a more active immune microenvironment. Despite this, immune evasion mechanisms remain a challenge, suggesting that combination therapies targeting both immune activation and suppression are necessary. Future research should focus on validating these findings and elucidating the role of tertiary lymphoid structures (TLS) in GBM. In contrast to adult brain tumors, pediatric brain tumors exhibit significant differences in immune composition, as highlighted by the study of Cao et al. This study uses single-cell RNA-sequencing (scRNA-seq) and bulk RNA-sequencing to comprehensively map the immune ecosystem in pediatric brain tumors, providing a deeper understanding of the TME and identifying potential therapeutic targets. The study suggests that targeting myeloid cells, rather than T cells, may be a more effective strategy for immunotherapy in these young patients. Importantly, the variability of the TME across different mouse models has been highlighted in the review by Carretta et al. Their work underscores the heterogeneity of cancer-associated fibroblasts (CAF) across various syngeneic mouse models, further illustrating the complexity of the TME. This variability suggests that selecting appropriate preclinical models and thoroughly understanding the specific characteristics of the TME are crucial for predicting therapeutic outcomes more accurately. Thus, researchers can develop more effective cancer treatments. Future research should focus on devising strategies to target CAFs and modulate the extracellular matrix (ECM), thereby enhancing the efficacy of immunotherapies in cancer treatment. Finally, the identification of predictive biomarkers could significantly enhance the precision of future studies, enabling us to identify patients who are most likely to benefit from the novel therapeutic strategies discussed above. The oncogene MYC, known as a central regulator of both cancer metabolism and immune evasion, plays a pivotal role in driving metabolic reprogramming that supports tumor growth and immune escape. Despite the challenges in directly targeting MYC, Venkatraman et al. focus on MYC interactome to uncover alternative targets and develop biomarkers that could predict responses to MYC-targeted therapies. Notably, they highlight the development of the MYC inhibitor OMO-103, which is currently in phase I clinical trials. The recently published results from this trial suggest that OMO-103 is a promising new therapy for targeting the MYC oncogene in solid tumors, demonstrating a favorable safety profile, preliminary efficacy, and the ability to inhibit MYC transcriptional activity (4). Future research should continue to explore the intricate interactions within the TME, develop personalized therapeutic strategies, and identify biomarkers that predict treatment response. This editorial brings together the significant findings from recent studies and outlines the future directions necessary to advance our understanding of the TME and its implications for cancer therapy.
